# Proteinaceous Residue Removal from Oat β-Glucan Extracts Obtained by Alkaline Water Extraction

**DOI:** 10.3390/molecules24091729

**Published:** 2019-05-03

**Authors:** Joanna Harasym, Ewa Żyła, Katarzyna Dziendzikowska, Joanna Gromadzka-Ostrowska

**Affiliations:** 1 Adaptive Food Systems Accelerator–Research Centre, Wrocław University of Economics, Komandorska 118/120, 53-345 Wrocław, Poland; 2 Department of Biotechnology and Food Analysis, Wrocław University of Economics, Komandorska 118/120, 53-345 Wrocław, Poland; 3 Department of Dietetics, Faculty of Human Nutrition and Consumer Sciences, Warsaw University of Life Sciences, Nowoursynowska 159c, 02-776 Warsaw, Poland; ewa_zyla@sggw.pl (E.Ż.); katarzyna_dziendzikowska@sggw.pl (K.D.); joanna_gromadzka_ostrowska@sggw.pl (J.G.-O.)

**Keywords:** oat, β-glucan, purification, protein, molar mass, molecular weight, starch, pancreatin, papain, amylase, amyloglucosidase

## Abstract

*Background*: Wet methods of 1-3, 1-4 -β-D-glucan isolation from cereals differ mainly in the type of grain fraction used as raw material, the solid-liquid ratio of β-glucan in raw material vs. solvent used, and the type of aqueous solvent modification (alkali, neutral or acidic). All these factors impact the characterization of the residues finally found in extracts. Oat bran is a rich source of globulin fraction which can be transferred into the extracts, especially when a high pH is employed. *Methods*: A multi-stage (enzymatic and acidic) purification procedure was performed to remove the residues, especially starch and protein, from β-glucan isolates from oat of different molar mass. Pancreatin, thermostable α-amylase, amyloglucosidase, and papain were used for consecutive residue removal. Three levels of low pH = 4.5, 3.5 and 3.0 were also tested for effective protein precipitation. *Results*: The starch hydrolysis and liquefaction significantly facilitate the proteinaceous matter removal although papain usage showed an intensive unfavorable impact on β-glucan molar mass. Soluble protein content was significantly decreased after pancreatin and α-amylase treatment, while the significant reduction of amine nitrogen was noted after complete starch hydrolysis and a second acidification step. *Conclusions*: A complex procedure employing different enzymes is needed to successfully reduce the possibly bioactive residues in isolated oat β-glucan fractions.

## 1. Introduction

1-3,1-4-β-D-Glucan has a very wide range of biological activities which reach far beyond the mechanistic impact of its viscosity in the gut [1,2,3,4]. Firstly recognized and approved for its cholesterol-lowering properties, the 1-3, 1-4- β-D-glucan recognition as a valuable resource has expanded impressively. As a glucose homopolymer with specific intramonomer linkages it must be perceived as a bioactive substance, which activity additionally depends on its molecular weight. 

Several studies have concluded that not only the high molecular mass fraction of purified oat β-glucan reveals bioactivity, but also the low molar mass fraction of this polymer triggers other metabolic pathways resulting in activities like antioxidative, anti-inflammatory properties in gastritis and enteritis, pro-apoptotic vs. human melanoma, antitumor activities vs. cancer cells: Me45, A431, and normal HaCaT and murine macrophages P388/D1, hepato- and gastroprotective activity, inflammation alleviation in gastritis, antitumor activities in human epithelial cancer or regulation of genes connected with the immune response [5,6,7,8,9,10,11].

Considering the reported specific bioactivity of this polymer its highly purified fractions should be studied, as the residual content of other active substances can modulate its own activity leading to misunderstanding of the metabolic path stimulation activity. It has been observed that in oat grain there are several compounds which can modulate metabolic activity on different pathways among which, in addition to β-glucan, phenolic molecules like ferulic acid, its derivatives, flavonoids, anthocyanins, and especially avenantramides, a group of phenolic molecules specific to oat, phospholipids, phytosterols, tocols, proteins and bioactive peptides are identified [12,13].

Highly purified oat β-glucan preparation from non-contaminated cultivars and known varieties are hard to find on the market, therefore many studies use self–developed procedures to isolate sufficient quantities for experiments. The resulting β-glucan thus have different contents of impurities of various origins which could distort the metabolic effect evaluation. The main problem related to in vivo studies regarding the activity of highly purified oat β-glucan fraction is thus the need for huge amounts of such ingredients to allow proper experiment planning.

Purified β-glucan can only be obtained by isolation from the plant matrix using different extraction techniques [14]. Wet extraction procedures can involve different extraction stage pHs like acidic [15,16], neutral [17,18,19] and alkaline [20,21,22,23] or the utilization of different enzymes for consecutive digestion of botanical fractions [24,25]. Despite the different characteristics of the process, alkaline water procedures deliver the highest yield of extracted β-glucan [22,23]. High pH is also known as very efficient conditions for the solubilization of both polysaccharides and proteins [26] therefore, the predominant contaminants are expected to be proteins and starch.

The majority of proteins in oat grain are saline-soluble globulins and alcohol-soluble prolamins [27], however, the globulins are the most abundant, reaching about 75% of the total oat grain protein [28], while the alcohol-soluble prolamins, called avenins, accounts only for 10 to 20% [29,30]. Depending from the oat grain fraction (whole groat, flour or bran) the different proteins can be prevalently extracted, which can further impact the characteristics of the peptides remaining in unpurified oat β-glucan fractions because subsequent isolation steps involve different pH levels. 

In silico methods have recently enabled the identification of bioactive peptides in cereal storage proteins mainly in oat and barley [31]. Sequences of angiotensin-I converting enzyme (ACE) inhibitors have been found as well as ones coding prolyl endopeptidase inhibitors and renin inhibitors. The potent ACE inhibitory peptides deriving from oat protein isolate hydrolysis have been confirmed in vitro [32]. The BIOPEP database was used which offers the possibility of “enzyme action” tool application on different protein sequences and when three oat protein sequences were verified this in silico method suggested that many enzymes like chymotrypsin C, pancreatic elastase, papain, ficin or thermolysin possess the capability to cut the oat proteins releasing bioactive peptides with antihypertensive activity [33]. 

It also has been proved that members of the amylase/trypsin inhibitors (ATI) family found in wheat and related cereals are potent triggers of an innate immune response, via activation of the toll-like receptor 4 (TLR4) on myeloid cells. The amylase/trypsin inhibitors activate the TLR4–MD2–CD14 path in vitro and in vivo after oral or systemic administration [34,35]. 

This bioactivity is characteristic for wheat, rye, and barley protein, but other cereals reveal significantly lower activity, which intensity can be grouped regarding respective wheat flour activity as lower than 20% for such raw materials as buckwheat, millet, and teff, lower than 10% for quinoa, and oats and lower than 2% for amaranth, rice, and corn.

Therefore, the main objective of this study was the evaluation of the possible procedure for a significant lowering of proteinaceous residuals content in oat β-glucan isolates of high- and low – molar mass obtained by alkaline-water extraction.

## 2. Results 

### 2.1. Proteinaceous Residuals Removal Characteristic Description

**Figure 1:**
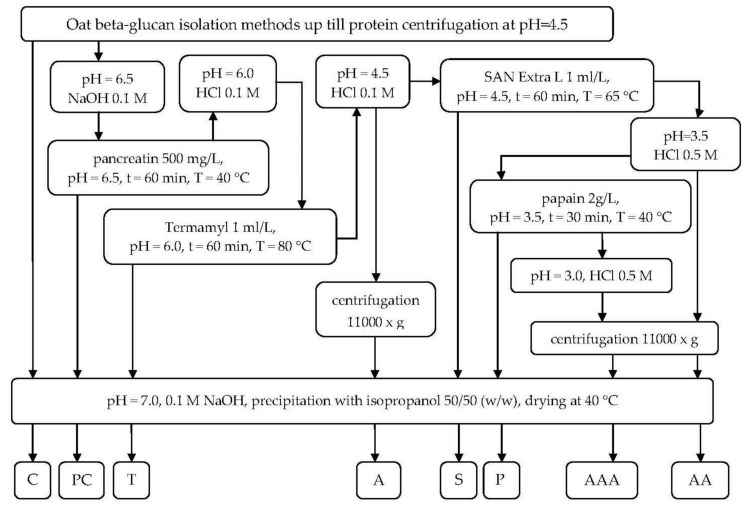
β-Glucan purification block-flow diagram. C—control sample, PC—sample after pancreatin digestion, T—sample after treatment with Termamyl SCDS, A—sample acidified to pH 4.5, S—sample treated with SAN Extra L, AA—sample acidified to pH 3.5, P—sample digested with papain, AAA - sample acidified to pH 3.0.

The purification steps were applied after reaching the processing stage of protein precipitation in pH = 4.5 according to the procedure described elsewhere [23]. After the primary oat β-glucan isolation procedures two wet streams were obtained—high- and low- molar mass β-glucan—from which two aliquots were taken as control samples and the remainder was further purified with different treatments. The combination of subsequent purification steps is presented in Figure 1. 

### 2.2. Starch and Glucose Content

The starch content in both types of β-glucan fractions was decreased after amylolytic digestion. The pancreatin enzymatic complex removed 41% and 39% of remaining starch in the high – (HM) and low- (LM) molar mass β-glucan fractions, respectively (Table 1). The thermostable α-amylase from *Bacillus licheniformis* was very efficient, reaching almost 83% reduction of remaining starch in a high molar mass fraction solution and 89% in a low molar fraction solution. After repetitive deproteination at pH = 4.5 the obtained fractions of both β-glucans were significantly purified from starch residues (up to 97%). Amyloglucosidase addition resulted in a non-detectable level of starch in further processed samples. Both fractions of β-glucan were efficiently destarched and observed differences were possibly due to the different viscosity of the treated solutions. Glucose content varied with the observed trend of subsequent dilutions and the precipitate of β-glucan as glucose is soluble in water: an isopropanol mixture, therefore most of sugar was removed with the solvent. The pancreatin activity differently reduced the glucose content in β-glucan of 7% and 35% in the HM and LM fractions, respectively. 

Further processing with Termamyl and acidification did not differ in glucose removal, which amount was finally decreased by 68% and 82% of the initial content in the control sample. After amyloglucosidase hydrolysis, the glucose content increased to 64% and 31% of initial control content and was further maintained regardless of the processing steps. The preserved glucose content in precipitated fractions of β-glucan could be probably due to sequestrating action of oligosaccharides precipitated also with beta-glucan, which while being dehydrated be polar alcohol start agglomerating in solution capturing smaller molecules within. Due to the absence of starch after amyloglucosidase treatment, the analysis of variance for the four steps was performed to assess the differences appearing in those stages. The two-factorial ANOVA revealed a strong impact of sample and treatment as well as their second order interrelation indicating the progressing changes in residual sugars with each stage of the process. The removal of viscosity-contributing compounds like starch or large dextrins resulted in a diluted solution capable of maintaining the compounds solubilized, which facilitates the selective precipitation of purified β-glucan fractions.

### 2.3. Protein Content

The proteinaceous matter content was dually assessed as soluble proteins and amine nitrogen content (Table 2). To avoid the overestimation, the protein factor was chosen to be 5.83 according to the FAO methods recommendation. 

The joint action of trypsin and chymotrypsin present in pancreatin mix resulted at 62% and 70% removal of soluble proteins content in β-glucan high- and low- molar mass fractions, respectively. However, the total amine nitrogen measurements revealed that insoluble proteinaceous matter was still present in the fractions, being only reduced by 38% and 19%, respectively. Further amylolytic treatment due to lowering of the solution viscosity allowed washing out more proteinaceous matter, leading to 71% and 87% removal of soluble proteins while amine nitrogen content was lowered to 44% and 51% in the high- and low molar mass fractions, respectively.

Subsequent acidic precipitation at pH = 4.5 significantly reduced the soluble protein content yielding an 11% and 27% of initial protein content in fractions compared to 17% and 28% of amine nitrogen content. The intensively liquefying and diluting action of amyloglucosidase resulted at further lowering of protein levels up till 99.4% of soluble moieties and 90.1% and 88.1% of amine nitrogen reduction. Further acidic precipitation step slightly reduced the amine nitrogen content reaching 93.4% and 99.5% purification level. The papain proteolytic activity allowed the removal of amine nitrogen to reach the level below the method precision, however in high-molar fractions some nitrogen still remained giving a positive protein content result.

### 2.4. Beta-glucan Content and Molar Mass

All the polymeric fractions strongly absorb water and swell in solution hindering further selective precipitation of other polymers, especially by dehydration, because such a mechanism is not specific. 

The primary procedure resulted in high- and low molar mass β-glucan fractions of 76.7% and 87.1% content of β-glucan, respectively (Table 3). After pancreatin digestion, the removal of starch and protein resulted in a 3% rise of β-glucan content in the high molar mass fraction of 2042.12 kD, while low molar mass β-glucan of 67.16 kD yielded 4.2% more. The original molar mass was significantly reduced only for the low-molar mass fraction, which could be the result of partial viscosity reduction due to the conjoint amylolytic and proteolytic activity of pancreatin. As molar mass was calculated indirectly from solution viscosity, it may be biased by the contribution of other polymers. The thermostable β-amylase hydrolysis resulted in a further rise of β-glucan purity of 14.4% and 8.2% in the high- and low- molar mass fractions, respectively, while the molar mass of β-glucan was not affected. Two subsequent purification stages slightly increased the values of β-glucan content reaching 97.5% and 99.25% for high- and low- molar mass fractions, respectively, without changing the molar mass in the high molar mass fraction and a slight decrease of the low molar mass fraction molar mass to 61.49 kD. Further purification revealed the very strong and undesired impact on molar mass. Although the purity of β-glucan was rising under the acidic conditions of pH = 3.5 and 3.0 this as well as papain digestion resulted in a massive diminishing of molar masses in both high- and low- molar mass fractions. The acidic conditions are known for their hydrolyzing impact on polysaccharides while several authors have also observed that papain possesses the β-glucanolytic activity which originates possibly from plant metabolic pathways or is the result of microbial contamination.

### 2.5. Possible Interactions between Compounds During Purification Process

The correlation analysis between parameters allows obtaining the deeper insight into their changes during particular stages as well as their mutual relations (Table 4). Pearson correlation analysis revealed strong positive interaction between starch and proteins, amine N and negative with β-glucan content in both fractions. β-Glucan was also strongly and negatively correlated with proteins and amine N content. The partial correlations coefficient indicates the linear relationship strength between the two variables, which have been firstly adjusted for their relationship to other variables. Therefore it can be useful for hidden relation observation. 

A strong negative correlation coefficient between amine N and β-glucan contents was noticed for both fractions, which can be caused by existing protein-polysaccharide linkages in the native matrix, which unbroken release is facilitated by the alkaline-water method and high pH of extraction. Also, a high positive correlation was observed for that coefficient in low molar mass fraction between amine N and molar mass, which can be the confirmation of possible amine nitrogen carrying moieties bonded to β-glucan chains.

## 3. Discussion

Among all wet extraction methods, the alkaline method is the most commonly reported one used for oat flour protein extraction [21,22,23]. The high extraction pH solubilizes more protein, however, it may reduce the protein nutritive value by encouraging the formation of lysinoalanine [36,37,38]. When the main purpose of extraction I β-glucan pure fraction recovery, the denaturing effect of high pH may be beneficial. The wet neutral and alkaline extraction procedures include extraction with carbonate buffer (pH = 10) at 60 °C; hot water extraction combined with starch hydrolysis at 90 °C; and extraction with sodium hydroxide solutions, among which the highest extraction yield of β-glucans > 90% was obtained from oat bran after 1.0 M NaOH usage [22].

The studies have also been performed on the extraction of oat β-glucan under acidic, alkaline, and enzymatic conditions and observed that the yield from acidic extraction was the lowest [15]. It was also reported that the acidic extraction of oat β-glucan led to a small amount of protein in isolates, as well as molecular weight was dependant on extraction temperature.

Simultaneously, at neutral and especially alkaline pH an increase in protein solubility was reported [26,37,38,39,40,41]. Such a phenomenon can be attributed to the charge increase of protein moieties and the increase of intra-chain repulsions, which can stretch intensively hydrogen bonds leading to their breaking and in consequences unfolding of polymer chains. 

Some researchers [40,42] have discussed the presence of phosphate residues observed in isolated oat β-glucan and suggested that protein-polysaccharide agglomerates covalently bonded which may contribute to oat β-glucan functionality bioactivity. To resolve the doubts Zielke et al. [41,43] employed the water extraction method at neutral pH without any alkali support and concluded no covalently bound proteins found in the β-glucan extracts produced by the method. Therefore, at least in oat β-glucan obtained from water extraction, there were no proteinaceous residuals influencing its bioactivity. However, in the investigation of wheat flour protein solubility, the maximum level was reached at pH = 10.5 [26], therefore the protein presence in alkali water extraction is obvious. 

β-Glucan fractions studied in this experiment was obtained from alkali extraction at 9.5 [23], therefore due to the raised net charge of proteins, a part of oat proteins (supposed to be globulins) was also extracted. Some purification procedure previously was applied by other researchers after protein precipitation in low pH but was not specifically targeted at protein content. Skendi at al. [17] isolated β-glucan from oat flour and precipitated protein from the acidification stage for 12 h at 25 °C, then rejected the sediment after centrifugation, neutralize it to pH = 7.0 and dialyzed it against distilled water for 3 days then freeze dry reaching protein content of 3–6.5%. Papageorgiou et al. [19] further developed the previous scheme adding centrifugation step after dialysis and precipitating β-glucan with 80% (*v*/*v*) ethyl alcohol, filter precipitated and washed it with 80% (*v*/*v*) ethyl alcohol, re-suspended in isopropanol and dried overnight at 40 °C. The authors didn’t determine the protein content but reached a β-glucan purity of 91.3%. Vasquez Mejia et al. [39,41] made an approach to evaluate the impact of subsequent purification steps on structural characterization of commercial and self extracted (pH = 8.0, 20% *w/v* CaCO_3_) barley β-glucans. The purification procedure consists of the amylolytic stage with 50 μL of thermostable α-amylase (3000 U/mL) incubation (35 min/95 °C), then cooling (60 °C), 100 μL of protease (50 mg/mL) addition and incubation (30 min/60 °C), repeated cooling and pH adjustment to 4.5 (HCl 5%) and 200 μL amyloglucosidase (140 U/mL) addition, followed by incubation at 60 °C/30 min. Finally precipitation was made with ethyl alcohol 99% (4:1, *v*/*v*), the precipitate was centrifuged (4850 g/4 °C/20 min) and washed again with ethyl alcohol 99% and finally freeze-dried. The designed multi-stage protein removal studied in this experiment was designed specifically for protein removal and to the authors’ best knowledge there is no comparative approach in the existing literature.

Oat (*Avena sativa* L.) represents a protein source with valuable nutrient properties. The oat grain is characterized by a better, than in most other cereals, the balance of the amino acids essential in the human diet [42,44]. The protein distribution in oat groat is different and such phenomenon impacts the extracts characteristic obtained from different milling fractions. In oat bran, protein content was higher than in oat flour [43,45] and oat bran is a good source of β-glucan or soluble fiber [44,45,46,47]. The oat is also perceived as less allergenic, and taking into account that allergenic activity is mainly connected with prolamin protein group, the described higher protein quality in oat grain may also be due to greater globulins accumulation (85% of globulin vs. 10–15% of prolamin). 

Oat globulin is rich in the amidated amino acids, glutamine and asparagine (ca. 20%) which strongly reflects its storage (also for nitrogen) role [27]. The structure analysis of oat globulin showed its hexameric composition of six subunits of about 55 kD. Acidic and basic separation of those polypeptides revealed differences both in sizes and isoelectric points [46,47,48,49], which were estimated to be 4–5 and 7–8, respectively [46,48]. Nieto-Nieto et al. [48,50] confirmed that the major proteins in oats have isoelectric points (pI) between 4.0 and 6.0. 

Prolamin fraction in oat is called avenin, which cross-reactivity by T cells induced by eating wheat was not found in vivo [49,51]. Additionally, it should be highlighted that none of the currently known wheat, barley, and rye epitopes was found in oats. In all oat varieties and species were found two avenin-specific epitopes [50,52] exist in all oat varieties and species [51,53], but oat intolerance is a seldom occurring case as very few CD patients react to these. In oat can be found different variants of wheat, barley, and rye epitopes, differing in two or three amino acids residues, what is supposed to block the T cell binding almost completely, depending on the position of the substitution in the nine-amino-acid T cell epitope [28]. However all these oat peptides are sensitive to pepsin, trypsin, and chymotrypsin digestion in the gastrointestinal tract, so they are not suspected to have any clinical relevance.

Therefore, it was reasonable to employ pancreatin as one of the enzymes used for purification from proteinaceous matter. Proteins from oats are beneficial; meanwhile, many applications have digested them with proteases to release peptides that will enhance their biological functions. In some studies globulin, hexameric structure (fraction > 300 kD) was degraded during treatment with alcalase or trypsin [52,54]. Recently, particular peptides were identified in HPLC fractions of hydrolyzed oat proteins with antioxidant and metal chelating properties [53,55]; it is also known that papain hydrolysates had better antioxidant activities because of its greater proteolytic action on most oat bran proteins. Therefore, the proteinaceous matter removal attempt was strongly justified.

Enzymatic pretreatment is frequently used for both cleaving the linkages within the polysaccharide matrix and liberate intercellular constituents, such as protein as well as for large extracted molecules hydrolysis to enlarge the extraction capacity of solvent leading to higher extraction efficiency. During enzyme assisted extraction many factors contribute to the final results as enzyme concentration, incubation time, temperature and pH, which additionally may affect either in an independent or interactive manner.

Guan and Yao [54,56] reported that the effects of two factors, namely pH and temperature, were the major contributors to protein extraction from oat bran. He also observed the decrease in viscosity of extraction solution caused by the degradation of β-glucan which was perceived by the authors as facilitating and enhancing protein extraction. Oat proteins digested with alcalase and trypsin were demonstrated to possess antioxidant activities [55,56,57,58], but trypsin hydrolysis is highly specific as trypsin is known to cleave peptides from the C-terminal end between lysine and arginine not followed by proline [57,59]. The oat globulin acidic polypeptide was found less susceptible to trypsin and remained the interior region of basic polypeptide intact [48,50,58,60]. Meanwhile, trypsin hydrolysis delivered 50–300 kDa fractions and the < 10 kDa fraction in higher amounts confirming that protein structures with higher molecular weight are not completely removed after trypsin hydrolysis. The difference observed in the results of this experiment between the amounts of soluble proteins and amine nitrogen irrespectively for fraction molar mass at control samples (5.85% vs. 6.16% 3.30% vs. 3.94%) and after pancreatin digestion (1.70% vs. 0.45% and 2.71% vs. 1.93%) confirmed that several of proteins lost their solubility after extraction stages or were covalently bonded to polysaccharides.

Immerstrand et al. [59,61] investigated the effects of various enzymes and extraction conditions on yield and molecular weight of β-glucans extracted from two batches of commercial oat bran manufactured in Sweden. They observed that protein hydrolysis also significantly reduced the peak molecular weight of β-glucans (by pancreatin to 908 × 10^3^ D and by papain to 56 × 10^3^ D). The molecular weight peak and distribution were assessed by high-performance size exclusion chromatography (HPSEC) with post-column addition of Calcofluor. It is logical and just recognized, that different isolation techniques impact the structural features of oat β-glucans [14]. Lazaridou et al. [18] after treatment with amylase and pancreatin removed the low molecular weight sugars and amino acids with dialysis obtaining the 90% yield and the average molecular weight was 203 × 10^3^ D or lower as measured by SEC/RI detector. The authors also observed the molecular weight of β-glucan reduction after α-amylase treatment from 2.8 × 10^6^ to 1.6 × 10^6^ while during papain treatment MW dropped from 1.6 × 10^6^ to 0.13 × 10^6^. They assigned the reduction in MW after amylasic digestion to a significantly lower amount of solubilized high molecular weight β-glucans, the heat treatment or by β-glucanasic contaminant enzymes from α-amylase. Also, Beer et al. [21] observed that β-glucan of MW of 1.8 × 10^6^ was obtained from oat bran treated with α-amylase while the yield was 64%. 

Although papain has been used previously to also purify β-glucans from oat bran [61] it must be confirmed that used enzyme preparation (from latex *Papaya carica*) has a strong cleaving action for high molecular weight β-glucans. The acid hydrolysis may also contribute to polysaccharides digestion [15] therefore from a certain pH level is to be expected the progressing decrease of molecular weight. Also, the same researchers using pancreatin mix observed only a drop in viscosity with only a 3% reduction after 3 h and an 18% reduction after 24 h. 

However, Autio et al. [62] using trypsin observed that viscosity of β-glucan samples was lowered, which could confirm existing interactions between protein and polysaccharides like β-glucan. The more pronounced action from the papain also may be allocated both to the residual β-glucanasic activity or strong cleavage action towards protein-β-glucan linkages. The resulting lower molecular weight may be the native one, actually existing in oat grain and usually overestimated during the investigation or may be the result of β-glucanolytic activity present in papain sample.

Amyloglucosidase usage facilitates the protein recovery due to dilution phenomenon which was confirmed by other researchers [61,63] where control (a sample from bran without an enzyme pre-treatment) had a protein content of 66.7% and in samples treated with amyloglucosidase the protein content rise to 83.8%. The amyloglucosidase application in this experiment seemed to be effective in polymers removal, as both starch and soluble proteins content had been significantly lowered. 

Oat globulins showed a limited solubility under acidic conditions. Researchers [62,64] observed the protein particles with hexameric structures at pH 7 whereas at pH 4 the protein structures were smaller than 20 kDa. In other works, the solubility of oat proteins has increased in highly acidic conditions (pH 1–4) [38,40,48,50] but, the present study did not consider pH beneath 3. However, the acidic conditions applied several times improved the protein removal, although its strong impact (especially at pH = 3.0) was detrimental for β-glucan molar mass which was reduced by almost 6–fold.

The Pearson correlation coefficient reveals the strong negative interaction between starch and beta-glucan and confirms that the main contaminant in purified beta-glucans fractions obtained from oat bran was starch. The positive correlations between starch and protein bodies suggest that the removal of starch supported the decrease of protein content both soluble and determined as amine nitrogen. The correlation is an effect of precipitation procedure, where simultaneously were precipitated proteins and starch. Such a phenomenon is confirmed by negative correlation factor between proteinaceous residuals and β-glucan content. The positive correlation observed between molar mass and amine N suggests the existence of protein moieties supposing links between β-glucan chains and can bias the accurate results of the molar mass determination. 

## 4. Materials and Methods

### 4.1. Oat Beta-Glucan Isolation

Oat β-glucan preparations of different molar masses were obtained with alkaline extraction of oat bran concentrate (OBC) having a β-glucan content of 20% (Microstructure, Warsaw, Poland). High molar mass fraction was isolated due to patented method [36,63] with minor modifications. Briefly, the 1 kg of OBC was heated in water/ethyl alcohol mixture (50:50 *w*/*w*) in 80 °C to remove the oat fat bodies and inactivate the endogenous enzymes. Then the solid fraction was separated and extracted in pH of 8.5 (NaOH in water, 1:40 *w*/*w*, solid:liquid ratio) to isolate β-glucan. The insoluble residues were removed with vibrating sieves and pH was lowered to 4.5 to precipitate the protein fraction which was further removed by centrifugation. The deproteinated supernatant was subjected to further enzymatic treatment. The β-glucan of low molar mass fraction was isolated using a patented method [37,64] as described in [22]. The raw material was frozen and extensively milled several times in the frozen state to efficiently reduce the particle size. Such prepared OBC was defatted in similar conditions as the high molar fraction and subsequently extracted in the same pH (8.5, NaOH in water, 1:40 *w*/*w*, solid:liquid ratio). Due to very small particles of the insoluble matter its removal with sieves was no longer possible. The remaining fraction was removed by centrifugation at 11,000 × *g* (MPW, Płock, Poland). The supernatant was deproteinated in pH = 4.5, protein sediment was removed with centrifugation and the obtained solution was processed.

### 4.2. Enzymatic Treatment for Residuals Removal

The solutions containing β-glucan of either high- or low- molar mass were divided each into two aliquots, the first one was adjusted with 0.1 M NaOH to pH = 7.0, precipitated with isopropanol addition, dried and analyzed as control sample (C), the remaining amount was subsequently processed with enzymes. 

#### 4.2.1. Pancreatin Digestion

The β-glucan-containing supernatants were adjusted with NaOH to pH 6.5 and 500 mg/L of pancreatin (in sodium phosphate buffer, pH 6.9) was added. Then the hydrolysis was conducted in 40 °C for 1 h. After hydrolysis the aliquot was removed, adjusted with 0.1 M NaOH to pH = 7.0, precipitated with isopropanol addition, dried and analyzed. The remaining sample was further processed.

#### 4.2.2. Termamyl Digestion

After pancreatin treatment the pH of solution was lowered to 6.0 (0.1 M HCl) and the heated up to 70 °C for Termamyl SCDS addition (1 mL/L, Novozymes A/S, Bagsværd, Denmark) with CaCl_2_ (70 mg/L), then maintained with stirring for 1 h. After hydrolysis the aliquot was removed, adjusted with 0.1 M NaOH to pH = 7.0, precipitated with isopropanol addition, dried and analyzed while the rest of the sample was subjected to acidification procedure. 

#### 4.2.3. pH 4.5 Precipitation

The solution was adjusted to pH = 4.5 with 0.1 M HCl and divided in half. One half was centrifuged in 9000 × *g* (MPW) and the sediment was discarded. The clear supernatant was neutralized to pH = 7.0 and β-glucan was precipitated with isopropanol. The remaining half was further processed.

#### 4.2.4. San Extra L Hydrolysis

Amyloglucosidase (San Extra L, Novozymes A/S) was added (1 mL/L) to the acidified (pH = 4.5) solution and temperature was maintained at 65 °C with stirring for 1 h. The sample was divided into two aliquots. First one was processed as usually to obtain β-glucan and the other one was subjected to the next stage of purification.

#### 4.2.5. pH 3.5 Precipitation

The solution was adjusted to pH = 3.5 with 0.1 M HCl and divided in two aliquots. One sample was centrifuged in 9000× *g* (MPW) and the sediment was discarded. The clear supernatant was neutralized to pH = 7.0 and β-glucan was precipitated with isopropanol. The other was digested with papain.

#### 4.2.6. Papain Digestion

Papain (2 g/L) was added in form of stock solution (10 mg/mL). Papain was initially dissolved in sodium phosphate buffer (0.1 M, pH = 6.0) containing 2.0 mM EDTA and 5.0 mM cysteine. The papain digestion was conducted in 40 °C with stirring for 0.5 h. The sample was divided into two aliquots as before, to obtain β-glucan and solution for further treatment. 

#### 4.2.7. pH 3.0 Precipitation

The solution was adjusted to pH = 3.0 with 0.1 M HCl and centrifuged in 9000× *g* (MPW) with cooling and the sediment was discarded. The clear supernatant was neutralized to pH = 7.0 and β-glucan was precipitated with isopropanol.

### 4.3. Analytical Methods

#### 4.3.1. Beta-glucan Determination

The (1,3) (1,4)-β-D-glucan content determination was performed according to AOAC 995.16 method using mixed linkage β-glucan assay kit (Megazyme International, Wicklow, Ireland) based on lichenase digestion. Due to the expected high concentration of β-glucan in analyzed samples the procedure of sample dissolution was prolonged to 30 min and supported with intensive mixing on low speed (IKA Werke, Staufen, Germany) to facilitate the sufficient hydration. The further steps of the procedure, especially enzymatic reactions were conducted without modifications.

#### 4.3.2. Starch Content Determination

Starch content in samples was performed according to AACC (American Association of Cereal Chemists) Method 76.13 with ready-to-use starch determination kit (Megazyme, Wicklow, Ireland) with some modifications. Briefly 50 mg of sample was used and the dilution sample volume was duplicated to avoid problems with viscosity of solution. Also dissolution of sample was prolonged to 30 min and supported with intensive stirring on a magnetic stirrer (IKA Werke, Staufen, Germany). 

#### 4.3.3. Glucose Determination

The residual glucose content was determined with the GOPOD reagent of a glucose assay kit K-GLUC according to the manufacturer’s procedure (Megazyme International, Wicklow, Ireland).

#### 4.3.4. Protein Content

The proteinaceous matter content was assessed dually with standard Kjeldahl and Lowry methods. Due to several processing stages which change both protein chains length and the solubility of sample it was justified to measure not only the soluble protein content. The total sum of nitrogen bound in organic substances as nitrogen in ammonia (NH_3_^-^N) and in ammonium (NH_4_^+^-N) is the suitable method for the efficient purification assessment. The protein conversion factor of 5.83 was applied for amine nitrogen assessed with Kjeldahl method. The Lowry method was proceeded using bovine serum protein as calibration curve standard.

#### 4.3.5. Molar Mass Determination

The mean molar mass determination was performed by intrinsic viscosity measurement. The viscosity of 0.5% (*w*/*v*) solution of β-glucan samples in Ostwald capillary viscosimeter was determined while the measurement was proceeded in 30 °C. The Mark-Houwink equation was applied to calculate the mean molar mass of the fraction.

### 4.4. Statistical Analysis

The results were taken in triplicate and reported as means with standard deviation. The analysis of variance (ANOVA) for mean values of was assessed with *p* < 0.05 significance level. Two factorial analysis of variance was performed to assess second order relation between sample and treatment for starch, glucose, beta-glucan and protein content. Pearson correlation coefficients and partial correlation coefficients were calculated between all the parameters with Statgraphics Centurion XVII (Bitstream, Cambridge, MA, USA) at a probability level of *p* < 0.05.

## 5. Conclusions

Proteinaceous matter removal from any grain extracts seems to be justified as the number of bioactivities connected with proteins or peptide presents in grain are constantly rising. Especially in case of oat β-glucan bioactivity evaluation it is reasonable to be aware of potential bioactivities coming from other bioactive compounds in oat. The isolated oat-β glucan is usually purified from other bioactive substances, as harsh extraction conditions (mainly alkaline, but also neutral or acidic) diminish the number of possible active protein moieties. But to be convinced of this polysaccharide bioactivity and due to scientific caution in case of existing modulators or triggers it is highly recommended to use the most purified β-glucan fractions possible.

## Figures and Tables

**Table 1:** Starch and glucose residual content in β-glucan fractions.

Sample	Starch (%/db)		Glucose (%/db)	
Treatment	HM	LM	HM	LM
C	15.55 ± 0.21 D, b	9.26 ± 0.35 D, a	3.70 ± 0.12 D, b	1.60 ± 0.14 C, a
PC	9.19 ± 0.89 C, b	5.69 ± 0.33 C, a	3.45 ± 0.03 D, b	1.05 ± 0.06 B, a
T	1.65 ± 0.07 B, b	0.65 ± 0.06 B, a	1.25 ± 0.01 A, b	0.35 ± 0.07 A, a
A	0.45 ± 0.04 A, b	0.25 ± 0.03 A, a	1.20 ± 0.14 A, b	0.30 ± 0.14 A, a
S	nd	nd	2.50 ± 0.14 BC, b	1.50 ± 0.28 C, a
AA	nd	nd	2.75 ± 0.02 C, b	1.15 ± 0.09 B, a
P	nd	nd	2.55 ± 0.04 BC, b	0.95 ± 0.03 B, a
AAA	nd	nd	2.40 ± 0.28 B, b	0.50 ± 0.14 A, a
Second order interactions *p* values				
Sample	***		***	
Treatment	***		***	
Sample x treatment	***		***	
C	15.55 ± 0.21 D	9.26 ± 0.35 D	3.70 ± 0.14 C	1.60 ± 0.14 C
PC	9.19 ± 0.89 C	5.69 ± 0.33 C	3.45 ± 0.07 C	1.05 ± 0.07 B
T	1.65 ± 0.07 B	0.65 ± 0.07 B	1.25 ± 0.07 A	0.35 ± 0.07 A
A	0.45 ± 0.07 A	0.25 ± 0.07 AB	1.20 ± 0.14 A	0.30 ± 0.14 A
S	nd	nd	2.50 ± 0.14 B	1.50 ± 0.28 C
Second order interactions *p* values				
Sample	***		***	
Treatment	***		***	
Sample x Treatment	***		***	

Mean values with different upper case letters imply significant differences between means in column at *p* < 0.05. Mean values with different lower case letters for the same parameter imply significant differences between means in row at *p* < 0.05: for second order interactions *** = *p* < 0.001, nd—not detected, d.b. per dry basis of solids weight, HM – high molar mass beta-glucan fraction, LM—low molar mass beta-glucan fraction, C—control type samples (without treatment), PC—samples after pancreatin digestion, T – samples after treatment with Termamyl SC DS, A—samples acidified to pH 4.5, S—samples treated with SAN Extra L, AA—samples acidified to pH 3.5, P – samples digested with papain, AAA - samples acidified to pH 3.0.

**Table 2:** Proteinaceous residuals content in obtained beta-glucan fractions.

Sample	Soluble Proteins (%/db)		Nitrogen x 5,83 (%/db)	
Treatment	HM	LM	HM	LM
C	5.85 ± 0.07 E, b	3.30 ± 0.14 D, a	6.16 ± 0.13 G, b	3.94 ± 0.04 F, a
PC	2.25 ± 0.21 D, b	1.00 ± 0.14 C, a	3.94 ± 0.04 F, b	3.21 ± 0.08 E, a
T	1.70 ± 0.14 C, b	0.45 ± 0.07 B, a	2.71 ± 0.04 E, b	1.93 ± 0.08 D, a
A	0.70 ± 0.14 B, b	0.09 ± 0.01 A, a	1.17 ± 0.08 D, a	1.14 ± 0.12 C, a
S	0.04 ± 0.01 A, a	0.02 ± 0.01 A, a	0.61 ± 0.04 C, b	0.47 ± 0.08 B, a
AA	0.01 ± 0.00 A	nd	0.41 ± 0.00 B, b	0.02 ± 0.01 A, a
P	0.01 ± 0.00 A, a	0.01 ± 0.00 A, a	0.03 ± 0.01 A, b	0.01 ± 0.00 A, a
AAA	0.01 ± 0.00 A	nd	0.01 ± 0.00 A	nd
Second order interactions *p* value				
Sample	***	***	***	***
Treatment	***	***	***	***
Sample x treatment	***	***	***	***
C	5.85 ± 0.07 E	3.30 ± 0.14 D	5.16 ± 0.13 E	3.94 ± 0.04 E
PC	2.25 ± 0.21 D	1.00 ± 0.14 C	3.94 ± 0.04 D	3.21 ± 0.08 D
T	1.70 ± 0.14 C	0.45 ± 0.07 B	2.71 ± 0.04 C	1.93 ± 0.08 C
A	0.70 ± 0.14 B	0.09 ± 0.01 A	1.17 ± 0.08 B	1.14 ± 0.12 B
S	0.04 ± 0.01 A	0.02 ± 0.00 A	0.61 ± 0.04 A	0.47 ± 0.08 A
Second order interactions *p* value				
Sample	***	***	***	***
Treatment	***	***	***	***
Sample x treatment	***	***	***	***

Mean values with different upper case letters imply significant differences between means in column at *p* < 0.05. Mean values with different lower case letters for the same parameter imply significant differences between means in row at *p* < 0.05: for second order interactions ** = *p* < 0.001, nd—not detected, d.b per dry basis of solids weight, HM—high molar mass beta-glucan fraction, LM—low molar mass beta-glucan fraction, C—control type samples (without treatment), PC—samples after pancreatin digestion, T—samples after treatment with Termamyl SC DS, A—samples acidified to pH 4.5, S—samples treated with SAN Extra L, AA—samples acidified to pH 3.5, P—samples digested with papain, AAA - samples acidified to pH 3.0.

**Table 3:** Proteinaceous residuals content in obtained β-glucan fractions.

Sample	β-Glucan (%/db)		Molar Mass (g/mol)	
Treatment	HM	LM	HM	LM
C	76.75 ± 0.21 A, a	87.10 ± 0.28 A, b	2054520 ± 77342.6 E	72260.0 ± 3258.4 E
PC	79.75 ± 0.92 B, a	91.35 ± 0.64 B, b	2042120 ± 73876.4 E	67166.0 ± 410.1 D
T	91.20 ± 1.27 C, a	95.30 ± 1.41 C, b	1776840 ± 31325.5 D	65877.5 ± 190.2 CD
A	95.40 ± 0.28 D, a	98.15 ± 1.06 D, b	1759600 ± 7030.8 D	61731.5 ± 2395.0 C
S	97.50 ± 0.42 E, a	99.25 ± 0.35 D, b	1705400 ± 142.8 D	61486.0 ± 2118.5 C
AA	98.20 ± 0.14 E, a	99.25 ± 0.35 D, b	987284 ± 1003.4 C	21100.5 ± 1101.0 B
P	98.05 ± 0.21 E, a	99.40 ± 0.14 D, b	752639 ± 5236.8 B	14888.0 ± 2531.4 A
AAA	97.95 ± 0.07 E, a	99.00 ± 0.14 D, b	333128 ± 17920.9 A	11924.0 ± 1303.9 A
Second order interactions *p* value				
Sample	***		***	
Treatment	***		***	
Sample x Treatment	***		***	
C	76.75 ± 0.21 A	87.10 ± 0.28 A	2054520 ± 77342.6 B	72260.0 ± 3258.4 C
PC	79.75 ± 0.92 B	90.35 ± 0.64 B	2042120 ± 73876.4 B	67166.0 ± 410.1B C
T	91.20 ± 1.27 C	95.30 ± 1.41 C	1776840 ± 31325.5 A	65877.5 ± 190.2A B
A	95.40 ± 0.28 D	98.15 ± 1.06 D	1759600 ± 7030.8 A	61731.5 ± 2395.0 A
S	97.50 ± 0.42 E	99.25 ± 0.35 D	1705400 ± 142.8 A	61486.0 ± 2118.5 A
Second order interactions *p* value				
Sample	***		***	
Treatment	***		***	
Sample x Treatment	***		***	

Mean values with different upper case letters imply significant differences between means in column at *p* < 0.05. Mean values with different lower case letters for the same parameter imply significant differences between means in row at *p* < 0.05: for second order *** = *p* < 0.001, nd—not detected, d.bper dry basis of solids weight, HM—high molar mass beta-glucan fraction, LM—low molar mass beta-glucan fraction, C—control type samples (without treatment), PC—samples after pancreatin digestion, T—samples after treatment with Termamyl SCDS, A—samples acidified to pH 4.5, S—samples treated with SAN Extra L, AA—samples acidified to pH 3.5, P—samples digested with papain, AAA - samples acidified to pH 3.0.

**Table 4:** Pearson correlation coefficient and partial correlation coefficient between parameters.

		Starch	Glucose	Proteins	Amine N	β-Glucan	Molar Mass
**Starch**	HM		-	0.96 ***	0.93 ***	−0.97 ***	-
	LM		-	0.95 ***	0.92 **	−0.97 ***	-
**Glucose**	HM	-		-	-	-	-
	LM	-		-	-	-	-
**Proteins**	HM	0.99 **	-		0.94 ***	−0.92 **	-
	LM	-	-		0.86 **	−0.92 **	-
**Amine N**	HM	-	-	0.95 *		−0.98 ***	0.79 *
	LM	-	-	-		−0.98 ***	0.79 *
**β-glucan**	HM	−0.97 *	-	0.98 *	−0.98 *		−0.71 *
	LM	-	-	-	−0.96 *		-
**Molar Mass**	HM	-	-	-	-	-	
	LM	-	-	-	0.98 *	-	

HM—high molar mass beta-glucan fraction, LM—low molar mass beta-glucan fraction, values in italics represent partial coefficients—*p* > 0.05; * *p* < 0.05; ** *p* < 0.01; *** *p* < 0.001.
